# Circulating extracellular vesicles and neutrophil extracellular traps contribute to endothelial dysfunction in preeclampsia

**DOI:** 10.3389/fimmu.2024.1488127

**Published:** 2024-12-13

**Authors:** Alex Ramos, Lina Youssef, Patricia Molina, Sergi Torramadé-Moix, Julia Martinez-Sanchez, Ana Belen Moreno-Castaño, Miquel Blasco, Elena Guillén-Olmos, Blanca De Moner, Marc Pino, Marta Tortajada, Marta Camacho, Maria Borrell, Francesca Crovetto, Maria Jose Ramirez-Bajo, Pedro Ventura-Aguiar, Elisenda Banon-Maneus, Jordi Rovira, Gines Escolar, Enric Carreras, Eduard Gratacos, Maribel Diaz-Ricart, Fatima Crispi, Marta Palomo

**Affiliations:** ^1^ Hemostasis and Erythropathology Laboratory, Hematopathology, Department of Pathology, Centre de Diagnòstic Biomèdic (CDB), Hospital Clínic de Barcelona, Institut d’Investigacions Biomèdiques August Pi i Sunyer (IDIBAPS), Universitat de Barcelona, Barcelona, Spain; ^2^ Barcelona Endothelium Team, Hospital Clínic de Barcelona, Institut d’Investigacions Biomèdiques August Pi i Sunyer (IDIBAPS), Barcelona, Spain; ^3^ BCNatal | Barcelona Center for Maternal Fetal and Neonatal Medicine, Hospital Clínic and Hospital Sant Joan de Déu, Institut d’Investigacions Biomèdiques August Pi i Sunyer (IDIBAPS), University of Barcelona, Barcelona, Spain; ^4^ Josep Carreras Leukaemia Research Institute, Hospital Clinic/University of Barcelona Campus, Barcelona, Spain; ^5^ Nephrology and Kidney Transplant Department, Center of Reference in Complex Glomerular Disease (CSUR), Hospital Clínic de Barcelona, Institut d’Investigacions Biomèdiques August Pi i Sunyer (IDIBAPS), Universitat de Barcelona, Barcelona, Spain; ^6^ Red de Investigación Cooperativa Orientada a Resultados en Salud RICORS2040, Universidad Autónoma de Madrid, Madrid, Spain; ^7^ Laboratori Experimental de Nefrologia i Trasplantament (LENIT), Institut d’Investigacions Biomèdiques August Pi i Sunyer (IDIBAPS), Barcelona, Spain; ^8^ Centre for Biomedical Research on Rare Diseases (CIBER-ER), Instituto de Salud Carlos III, Madrid, Spain; ^9^ Hematology External Quality Assessment Laboratory, Biomedical Diagnostic Center, Hospital Clinic of Barcelona, Barcelona, Spain

**Keywords:** pre-eclampsia, exosome, neutrophil activation, endothelium, complement membrane attack complex, oxidative stress

## Abstract

**Background:**

Preeclampsia (PE) is a pregnancy complication characterized by hypertension, proteinuria, endothelial dysfunction, and complement dysregulation. Placenta-derived extracellular vesicles (EVs), necessary in maternal–fetal communication, might contribute to PE pathogenesis. Moreover, neutrophil extracellular traps (NETs) play a pathogenic role in other complement-mediated pathologies, and their contribution in PE remains unexplored.

**Materials and methods:**

EVs were isolated from PE (peEVs) and normotensive pregnant women sera. NETs were obtained incubating donor-pre-activated neutrophils with PE or control sera. Microvascular (HMEC) endothelial cells (ECs) were incubated with PE or control sera with or without (depleted sera) EVs or NETs, to assess changes in VCAM-1, ICAM-1, VE-cadherin, eNOS, VWF, ROS, and C5b-9 deposits. Results were expressed as fold increase vs. control.

**Results:**

VWF, VCAM-1, and ROS expression was significantly higher in cells exposed to PE sera vs. control (12.3 ± 8.1, 3.6 ± 2.3, and 1.8 ± 0.2, respectively, *p* < 0.05), though significantly lower in cells exposed to depleted PE (dPE) sera (6.1 ± 2.7, 0.7 ± 0.6, and 1.2 ± 0.1, respectively, vs. control, *p* < 0.05). EC exposure to depleted control sera supplemented with peEVs (dC+peEVs) significantly increased VWF, VCAM-1, and ROS compared to non-supplemented sera (4.5 ± 0.3, 2.8 ± 2.0, and 1.4 ± 0.2, respectively, *p* < 0.05). ICAM-1, VE-cadherin, and C5b-9 did not differ among groups. ECs incubated with PE-NETs increased VWF and VCAM-1 and decreased VE-cadherin expression vs. control (4 ± 1.6, 5.9 ± 1.2, and 0.5 ± 0.1, respectively, *p* < 0.05), and notably increased C5b-9 deposit (7.5 ± 2.9, *p* < 0.05). ICAM-1 and ROS did not differ.

**Conclusions:**

Both circulating EVs and NETs from PE pregnant women exhibit a deleterious effect on ECs. Whereas EVs trigger a pro-oxidant and proinflammatory state, NETs potentiate the activation of the complement system, as already described in PE.

## Introduction

Preeclampsia (PE) is a pregnancy-specific complication that affects 2%–8% of all pregnancies and is the leading cause of maternal and neonatal mortality and morbidity. PE is characterized by new-onset hypertension after 20 weeks of gestation that is usually accompanied by proteinuria (1, 2). Although the etiology of this multifactorial disease remains unclear, endothelial dysfunction, complement dysregulation, and the imbalance of angiogenic factors have been postulated as key elements of this complication (3, 4).

Extracellular vesicles (EVs) originated from placental trophoblast are increasingly released into the maternal circulation. EVs contain RNAs, lipids, proteins, and DNA and play a key role in endocrine and paracrine communication in both physiologic and pathologic pregnancies (5). In PE, an increment of EVs has been reported. PE-EVs contain phosphatidylserine in their surface, resulting in widespread blood clot formation (6) and fibrin depositions (7), and contributing to a PE hypercoagulation state. Moreover, PE-EVs exhibit an increase of tissue factor (8, 9), which is involved in the activation of monocytes, macrophages, and the vascular endothelium (10).

These EVs are strongly related with NET formation as they could directly activate neutrophils leading to NETosis (11). Moreover, EVs interact with maternal immune cells (12) through fusion protein syncytin-1 and promote the release of proinflammatory cytokines [such as IFN-γ, interleukin (IL)-8, IL-12, and TNF-α] (13), which, in turn, induces neutrophil activation and the subsequent NET release. Finally, the ischemic situation occurring in PE placenta increases the production of reactive oxygen species (ROS) (14), which also contributes to the exacerbation of NETosis, increasing not only NETs’ soluble levels (15) but also NETs’ deposits on placenta (11). At the same time, the excessive production of NETs and their deposit on placenta hinders trophoblast migration and could contribute to defective placental development, scarce perfusion, and increased inflammation together with EV release (16).

NETs, composed of extracellular strings of DNA, histones, and enzymes such as elastase and myeloperoxidase, play an important role in the elimination of pathogens. The presence of circulating NETs has been described in healthy and pathologic pregnancies (17) and also in association with endothelial dysfunction (18), as extracellular histones activate NF-kB and the transcription of activator protein 1 (AP-1) via Toll-like receptor in vascular cells (19, 20), increasing the production of inflammatory cytokines and the expression of tissue factor favoring platelet activation and aggregation (16). Cytokines released in PE together with the C5a component of the complement system contribute to the upregulation of TLRs in neutrophils and the consequent NET release (21). This NET production increase (22) contributes to widespread damage of the maternal endothelium in PE (23) causing multiorgan failure, and especially affecting both liver and kidneys (24), together with innate immune system dysregulation.

Although the complement system activation is normal in pregnancy to protect against pathogens and to facilitate the clearance of placental debris (25), the overactivation of the complement system has been related to the severity of the PE and to the development of PE and adverse pregnancy outcomes (26–28). This overactivation of the lytic complex was demonstrated by our group in an *in vitro* model exposing endothelial cell (EC) culture to activated plasma from PE pregnancy (29).

Together with the complement system activation, the endothelial damage has also been associated with PE (30), the pathophysiological mechanisms that connect these entities remain unknown. Therefore, the aim of the present study was to investigate the potential role of both EVs present in the sera of PE pregnant women and NETs as inductors of endothelial damage and the complement dysregulation occurring in PE. We hypothesize that the bidirectional relationship between the two components creates a vicious cycle that contributes to the clinical manifestations of PE increasing the proinflammatory and procoagulant state of the endothelium. The knowledge of the mechanisms involved may improve the management of these pregnancies by providing more targets for future therapeutic strategies.

## Materials and methods

### Study population

A total of 58 singleton pregnancies were prospectively enrolled for this study in the Department of Maternal–Fetal Medicine at Hospital Clinic, Barcelona, Spain, between 2016 and 2021. The study population comprised blood samples from two groups: normotensive mothers considered as controls (*n* = 23), and pregnancies complicated by PE (*n* = 35). PE was defined as systolic blood pressure >140 mmHg and/or diastolic blood pressure >90 mmHg on two occasions, at least 4 h apart, and proteinuria (>300 mg/24 h or protein/creatinine ratio > 300 mg/g) developed after 20 weeks of gestation (31–33). Mothers under 18 years, twin pregnancies, congenital malformations, chromosomal anomalies, and intrauterine infections were excluded. Gestational age was calculated based on crown–rump length at the first-trimester ultrasound. This study was approved by the ethics committee of the Hospital Clinic (HCB/2020/0240) and conformed to the ethical guidelines of the Helsinki Declaration. All participants provided informed written consent before sample collection.

### Sample collection and storage

Maternal blood samples were drawn at the time of diagnosis or at matched gestational age for controls and collected into citrated and non-anticoagulated tubes. Plasma and sera were separated by centrifugation at 1,500*g* for 10 min at 4°C, filtered through a 0.22-μm filter, and stored at −80°C until further use. All samples were enrolled in the National Register of Biobanks for biomedical research that conformed to Real Decree 1716/2011.

### Study design

To explore EV and NET contribution to the endothelial damage associated with PE, an *in vitro* endothelial dysfunction model was used. To evaluate the role of EVs, ECs in culture were exposed to four different conditions: a pool of control sera (C), a pool of PE sera (PE), a pool of PE sera depleted from EVs (dPE), and a pool of control sera depleted from control EVs and supplemented with EVs from PE (dC+peEVs). Then, changes in different biomarkers of endothelial activation and damage were assessed. NETs were obtained from healthy pre-activated neutrophils exposed to control or PE sera (C-NETs and PE-NETs, respectively). ECs in culture were exposed to NETs to evaluate their effect on endothelial damage biomarkers. The effect of both peEVs and NETs on C5b-9 deposits on ECs was explored.

### EV isolation

EVs and depleted serum were obtained from sera pools of patients in the study groups by differential centrifugation (34). Briefly, sera samples were centrifuged at 800*g* for 7 min and at 2000*g* for 15 min to remove cell debris. Subsequently, the supernatants were filtered through a 0.22-µm pore filter and ultracentrifuged (Optima L100XP, Beckman) at 100,000*g* for 2 h. Then, sera depleted from EVs were recovered and the EVs were subsequently washed with PBS and followed for a second ultracentrifugation. Pellets were suspended in PBS and stored at −80°C until further use.

### EV nanoparticle tracking analysis

The size distribution and concentration of EVs were both measured using a NanoSight NS300 (Malvern Instruments Ltd., Malvern, UK), equipped with a 488-nm laser and camera—High-sensitivity sCMOS. Samples were diluted with PBS. For each sample, 5 videos of 50 s at camera level 10 and threshold 5 were captured. Analysis was done with nanoparticle tracking analysis (NTA) 3.4 Analytical software. Sample contamination was discarded as any sample showed the characteristic fog pattern of contaminated samples.

### EV study by electron microscopy

EV characterization by electron microscopy was performed using a holey carbon support film on a 400-mesh copper grid. Three microliters of the EV sample was placed on a plunger (Leica EM GP). The suspension was vitrified by rapid immersion in liquid ethane (−179°C), and the grid was mounted on a Gatan 626 cryo-transfer system and inserted into the microscope. The images were taken using a cryo-electron microscope operating at 200 kV, recorded on a GatanUltrascan US1000 CCD camera, and analyzed with a Digital Micrograph 1.8 (*n* = 3 per group).

### Flow cytometry analysis of EVs

The phenotypic characterization of EVs isolated from sera pools from patients included in the study and used in *in vitro* studies was done using the MACSPlex Exosome Kit (Miltenyi Biotec, Bergisch Gladbach, Germany) following the manufacturer’s recommendations. This kit enables the simultaneous detection of 37 surface epitopes (CD3, CD4, CD19, CD8, HLA-DR, CD56, CD105, CD2, CD1c, CD25, CD49e, ROR1, CD209, CD9, SSEA4, HLA-BC, CD63, CD40, CD62P, CD11c, CD81, MCSP1, CD146, CD41b, CD42a, CD24, CD86, CD44, CD326, CD133/1, CD29, CD69, CD142, CD45, CD31, CD20, and CD14) that are known to be present on different vesicles plus two isotype control beads (REA and IgG1). Briefly, 1 × 10^9^ EVs (quantified by NTA) were diluted in 120 µL of MACSPLex buffer with 15 µL of beads and then were incubated under gentle agitation and protected from light on a rotor overnight at 4°C. After incubation and washing steps, APC antibodies against CD9, CD63, and CD81 were added and incubated for 1 h at RT under gentle agitation and protected from light. The samples were washed and detected using a BD LSRFortessaSORP cytometer analyzer (BD Bioscience, NJ, USA). MFI values of buffer control were subtracted, and subsequently, the MFI value of samples was normalized to the median fluorescence intensity of CD9/CD63/CD81. Data analysis was performed with FACS DIVA software (BD Biosciences, Heidelberg, Germany).

### NET isolation

Citrated blood from a healthy human donor was mixed in the same proportion with Polymorphprep (Progen Biotechnik GmbH, Heidelberg, Germany) and centrifuged (500*g*, 35 min at RT) to obtain neutrophils. Then, isolated neutrophils were centrifuged with 25 mL of HBSS medium without Ca^2+^ and Mg^2+^ (400*g*, 10 min at RT) to obtain a pellet that was mixed with a hypotonic lysis solution (1 mL of H_2_Od + 0.33 mL of NaCl 3.6% + 20 mL of HBSS medium without Ca^2+^ and Mg^2+^) to remove red blood cells (250*g*, 5 min at RT). This pellet was resuspended with Ca^2+^ and Mg HBSS medium to obtain 1 × 10^7^ neutrophils/mL.

Then, NET production was performed following Schreiber et al.’s protocol (35): 1 mL of 1 × 10^6^ neutrophils/mL was seeded on pretreated coverslips (incubated with 2 mL of poly-L lysine 0.01% for 15 min at 37°C and 5% of CO_2_). After 15 min, 4 μL of TNFalpha 2 ng/mL was added to preactivated neutrophils and the coverslips were incubated for 15 min (37°C, 5% CO_2_). Then, neutrophils were exposed to 20 μL of 1/1,000 stock PMA (10 mg/μL) as positive control, to 500 μL of control sera from normotensive pregnant women (to obtain C-NETS), or to 500 μL of PE sera (to obtain PE_NETS) (3 h, 37°C, 5% CO_2_). Finally, coverslips were washed with HBSS medium without Ca^2+^ and Mg^2+^, incubated with 500 mL of DNase solution (20 UI/mL) for 30 min, and scratched to obtain NETs.

Coverslips of each group were stained with SYTOX green (2 μL of 1/100 of SYTOX green + 1 mL of HBSS medium without Ca^2+^ and Mg^2+^, for 10 min, RT). Then, fluorescence was evaluated by light microscopy as previously described (36). The results were expressed as the percentage of the area covered by NETs (mean ± SD). Additionally, DNA concentration of the obtained NETs was measured in supernatants, after scratching the coverslips, by a nanodrop (ND-1000, Thermo Scientific).

### Endothelial cell culture

Human microvascular endothelial cells (HMEC from ATCC, Manassas, USA) were grown with medium MCDB131 (Gibco-BRL, Madrid, Spain), supplemented with fetal bovine serum (Biowest, Nuaillé, France), L‐glutamine, penicillin/streptavidin (Gibco-BRL, New York, USA), endothelial growth factor (BD Biosciences, Erembodegem, Belgium), and hydrocortisone (Sigma-Aldrich, Madrid, Spain). Microvascular (HMEC) ECs were maintained at 37°C in a CO_2_ atmosphere (5%) and used at passages 5–12. ECs were seeded on pretreated 18×18 mm^2^ coverslips, in 6‐well plates (VWR, Radnor, USA). After 24 h, cells were exposed to the different conditions under study (for 48 h).

### VWF, VCAM-1, ICAM-1, eNOS, and VE-cadherin expression in endothelial cells exposed to EVs and NETs

To evaluate the expression of VWF, VCAM-1, ICAM-1, VE-cadherin, and eNOS, ECs were exposed to EV groups (C, PE, dPE, dC+peEVs) and media containing 20% of NETs solution (C-NETs or PE-NETs). Then, cells were fixed with 4% paraformaldehyde (for 10 min), blocked with 2% BSA and incubated with a primary antibody against: VWF 1:2,000 (Dako/Agilent, Santa Clara, USA); VCAM-1 1:100 (GeneTex, Irvine, USA); ICAM1 1:50 (SantaCruz Biotech, Dallas, USA); eNOS dilution 1:50 (SantaCruz Biotech, Santa Cruz, USA), and VE-cadherin 1:500 (GeneTex, Irvine, USA), (1 h, RT) and a secondary antibody IgG conjugated with Alexa 488 or 594 (Molecular Probes, Eugene, USA) (dilution 1:500 for Alexa 488 and dilution 1:2,000 for Alexa 594), 1 h, at RT, and 4′,6-diamidino-2-phenylindole. Then, fluorescence was evaluated by light microscopy as previously described (29). The results were expressed in fold increase vs. control.

### Reactive oxygen species production in endothelial cells exposed to EVs and NETs

Changes in the production of ROS were explored by immunofluorescence. EC seeded coverslips were preincubated with ROS detection reagent CM.H2DCFDA (Molecular Probes, New York, USA) at 37°C for 30 min. After three PBS washes, ECs were exposed to the different EV conditions and both NET groups (37°C, 30 min). ROS production was monitored by fluorescence microscopy (Leica DM4B, Barcelona, Spain) and 15 images of each sample were randomly captured through a video camera (Leica DFC9000GT, Barcelona, Spain). The fluorescence intensity of the images was analyzed by FIJI software (ImageJ Fiji, 10 Bethesda, Rockville, USA). The results were expressed in fold increase vs. control.

### C5b-9 deposition on endothelial cells exposed to NETs obtained from healthy pregnancies or preeclampsia

To evaluate C5b-9 deposition, a previous protocol was used (37), activated plasma was obtained by adding control sera to control citrated plasma (1:1), and then 6 μL of EVs or 10% of NET solution was added (*n* = 3 and *n* = 5, respectively). The area covered by the C5b-9 deposit was calculated and expressed as the average fold increase of each condition versus control.

### Statistical analysis

Data normality was checked, and then parametric or non-parametric test was applied. Scheffe test was performed for homogeneity parametric results and *post-hoc* Games-Howell was carried out for non-homogeneity parametric results. Non-parametric results were evaluated by median test. Results were expressed as fold increase (mean ± SD) and differences were considered statistically significant when the *p*-value was <0.05.

## Results

### Baseline and perinatal characteristics of the study populations

Baseline characteristics of the study populations are summarized in **Table 1**. Maternal characteristics were similar between the two study groups. However, significant differences were detected in some parameters: PE pregnant women showed a higher body mass index compared to controls, and gestational age at delivery was earlier in 26 of 35 PE mothers (preterm deliveries). Furthermore, cesarean section was needed in 80% of PE deliveries, a significantly higher proportion compared to controls. In addition, the median birthweight of PE fetuses was below the 10th percentile with more than half of these fetuses having FGR (68.6%). To note, six PE patients developed HELLP syndrome.

**Table 1:** Baseline and perinatal characteristics of the study populations.

	Controls n = 23	Preeclampsia n = 35
Maternal characteristics		
Age (years)	34 (30.8–37.9)	34.9 (30.4–38.2)
Ethnicity		
White	13 (56.5)	23 (65.7)
African	1 (4.4)	3 (8.6)
Latin	5 (21.7)	4 (11.4)
Asian	4 (17.4)	5 (14.3)
Pre-gestational body mass index (kg/m^2^)	21.3 (19.2–22.7)	25.1 (21.2–29.1)*
Nulliparity	12 (52.2)	15 (42.9)
Use of assisted reproductive technologies	0 (0)	6 (17.1)*
Smoking during pregnancy	2 (8.7)	3 (8.8)
Perinatal outcomes		
Gestational age at delivery (weeks)	40 (38.9–41)	34.1 (32.4–37.1)*
Preterm delivery#	1 (4.3)	26 (74.3)
Cesarean section	4 (17.4)	28 (80)*
Female gender	13 (56.5)	14 (41.2)
Birthweight (g)	3,262 (3,020–3,418)	1,778 (1,486–2,450)
Birthweight centile	38 (20–51)	2.5 (0–23)
Fetal growth restrictionΨ	0 (0)	24 (68.6)*
APGAR score 5 min <7	0 (0)	10 (28.6)*
Umbilical artery pH	7.19 (7.13–7.24)	7.22 (7.17–7.26)
Stillbirth	0 (0)	1 (2.9)

Data are median (interquartile range) or *n* (%) as appropriate.

#Preterm delivery defined as delivery occurring before 37 weeks of gestation.

ΨFetal growth restriction defined as birthweight below the 10th centile according to local standards.

*p < 0.05 by Mann–Whitney *U* test, Pearson χ^2^ or Fisher exact tests as appropriate, compared to controls.

### Differential composition of PE-EVs compared to control EVs

**Figure 1:**
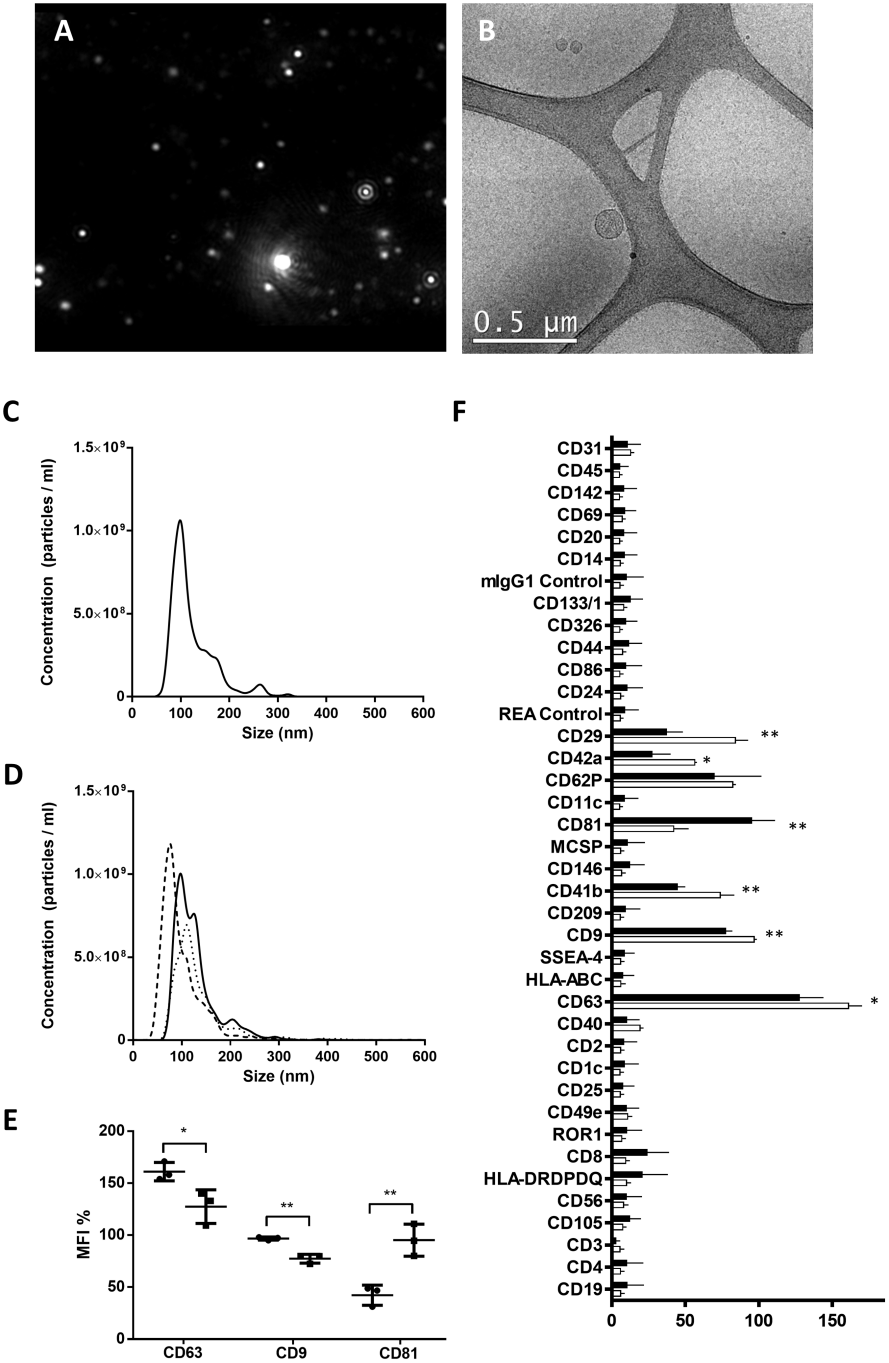
Characterization of EVs isolated from control and preeclampsia pool serums. Upper images show EV characterization by **(A)** nanotracking analysis with the absence of the characteristic fog pattern of contaminated samples and **(B)** electron microscopy (scale bars, 0.5 μm). Cryo-electron microscopy allows one to visualize the grid with an irregular distribution of hole sizes and shapes containing EVs of various sizes. **(C, D)** NTA particle concentration and size distribution of one control pool and three preeclampsia sera pools, respectively. **(E)** MFI of EV-markers CD9, CD63, and CD81 (control in black and PE in blue), obtained from MACSPlex analysis of EVs surface markers, and **(F)** phenotypic signature of EVs quantified by the MACSPlex Exosome Kit in conjunction with flow cytometry. Black bars correspond to PE pools and white bars correspond to control pools. *p <  0.05 and **p <  0.01 compared to the control group.

Through NTA and electron microscopy techniques, both presence of EVs and non-contamination of the samples were assessed (**Figures 1A, B**). No differences were observed regarding the concentration and the size of the PE and control EVs (**Figures 1C, D**). However, PE and control EVs characterized by MACSPlex exosome kit in conjunction with flow cytometry showed significant differences in their composition. PE-EVs showed a statistically significant decrease in the expression of CD63, CD9, CD29, CD42a, and CD41b compared to control EVs (CD63, CD29, and CD41b, *p* < 0.05; CD9 and CD42a, *p* < 0.01), and an increase in CD81 expression (*p* < 0.01) (**Figures 1E, F**).

### EVs from PE pregnancies contribute to endothelial damage

**Figure 2:**
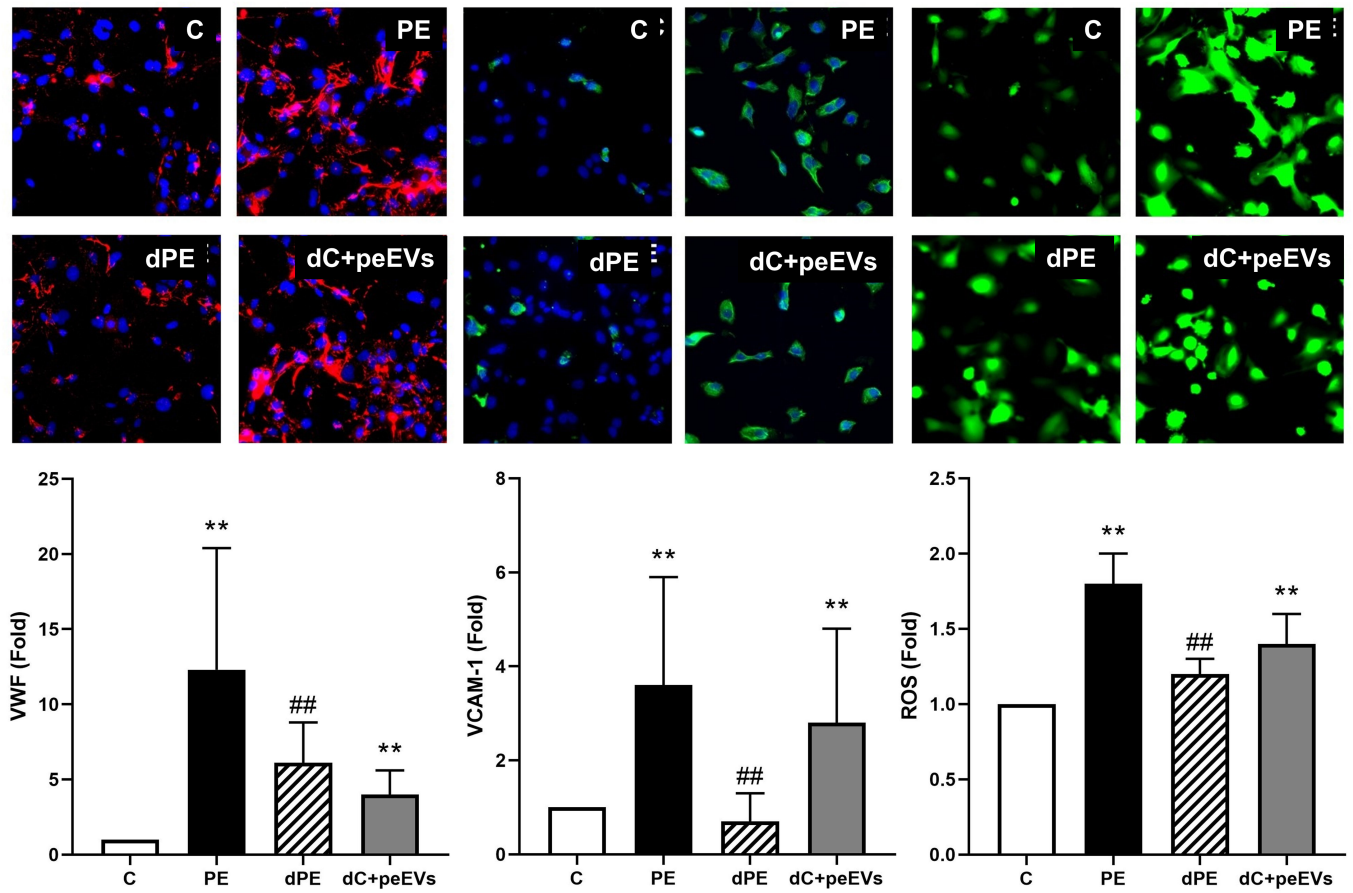
The exposure of endothelial cells to PE-EVs increased the expression of dysfunction endothelial markers. Representative microscopy image (40×) of VWF, VCAM-1, and ROS (red: VWF; ROS: green; and VCAM-1: green) on endothelial cells (4′,6-Diamidino-2-phenylindole-stained nuclei, blue) induced by exposure (48 h) to control, preeclampsia, depleted preeclampsia (without PE-EVs), and control sera pool supplemented with PE-EVs. The bar graph indicates the average fold increase of the different conditions compared to control. The vertical line indicates the standard deviation. **p < 0.01 compared to the control condition, ##*p* < 0.01 compared to the PE condition.

**Figure 3:**
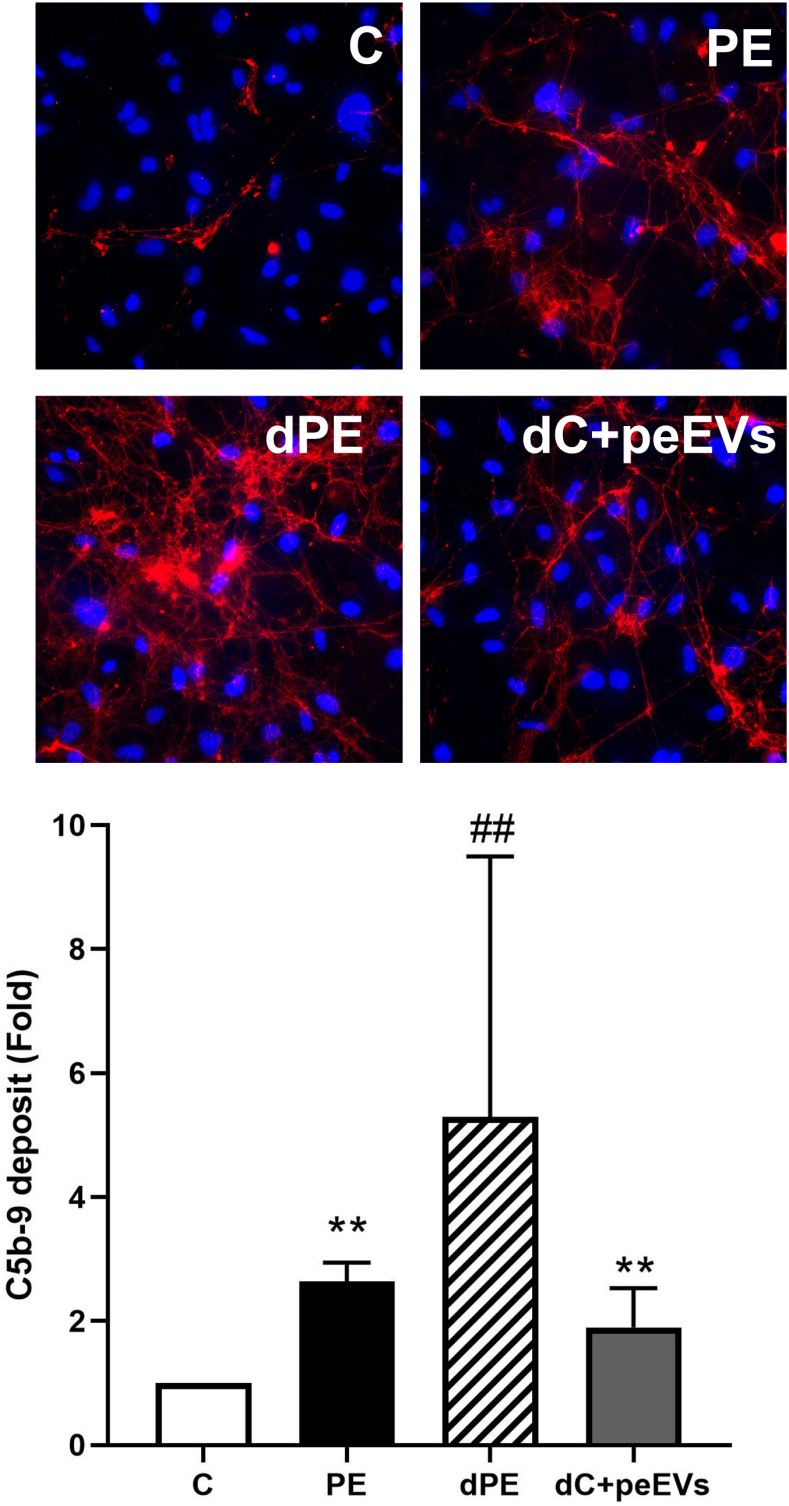
The complement system dysregulation is not mediated by EVs. Representative microscopy image (40×) of C5b-9 deposit (red) on endothelial cells (4′,6-diamidino-2-phenylindole-stained nuclei, blue) induced by exposure (4 h) to control, preeclampsia, depleted preeclampsia (without PE-EVs), and control sera pool supplemented with PE-EVs. The bar graph indicates the average fold increase of the different conditions under study compared to control. The vertical line indicates the standard deviation. **p < 0.01 compared to control, ##*p* < 0.01 compared to PE.

As summarized in **Figure 2**, the exposure of ECs to PE sera induced an increase of VWF and VCAM-1 expression and ROS induction compared to control sera (fold change of 12.3 ± 8.1, 3.6 ± 2.3, and 1.8 ± 0.2, respectively, *p* < 0.05). Furthermore, these biomarkers (VWF, VCAM-1, and ROS) were significantly lower in ECs exposed to depleted PE sera with respect to ECs exposed to PE sera (fold change of 6.1 ± 2.7 vs. 12.3 ± 8.1, 0.7 ± 0.6 vs. 3.6 ± 2.3, and 1.2 ± 0.1 vs. 1.8 ± 0.2 respectively, *p* < 0.05). Finally, the exposure of ECs to control depleted sera supplemented with pdEVs from PE significantly increased VWF, VCAM-1, and ROS with respect to control sera (fold change 4.5 ± 0.3, 2.8 ± 2.0, and 1.4 ± 0.2, respectively, *p* < 0.05). No differences were observed between groups for eNOS, ICAM-1, VE-cadherin (**Supplementary Figure 1**), and C5b-9 deposition (**Figure 3**).

### PE sera increase NET production by neutrophils from a donor compared to control sera exposure

**Figure 4:**
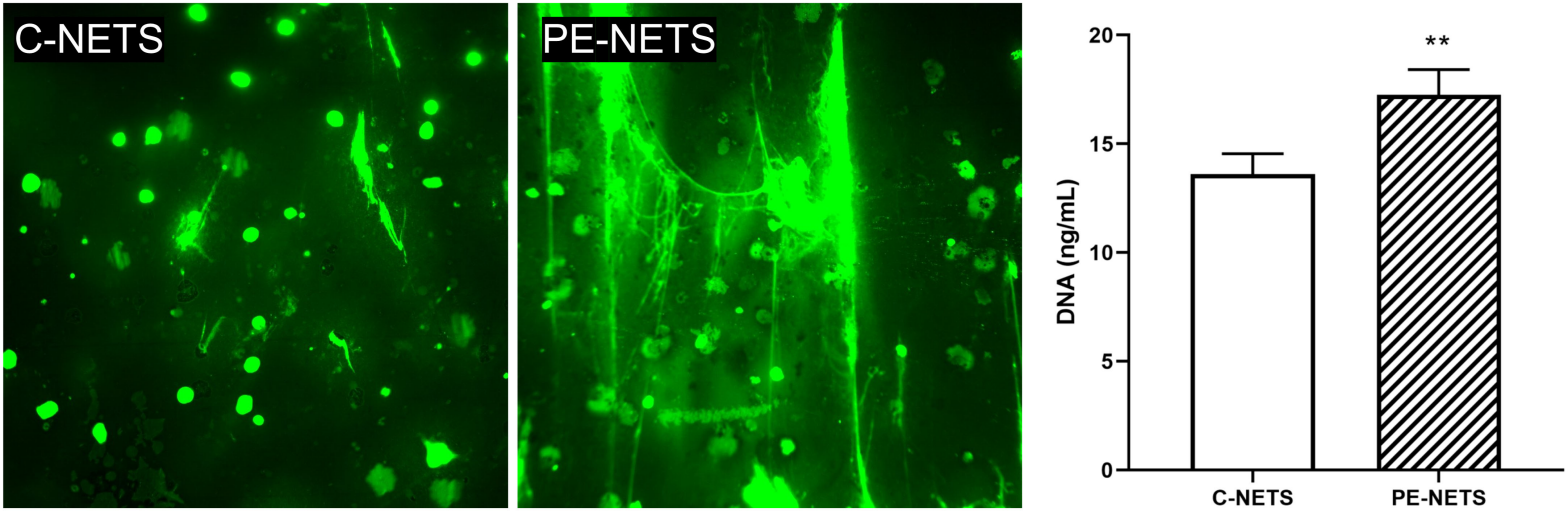
PE sera induce NET generation in neutrophils from a healthy donor. Micrographs of SYTOX green staining showed an increase in NET production by isolated donor neutrophils preactivated with TNFalpha incubated with PE sera compared to preactivated donor neutrophils incubated with sera from healthy pregnant women (micrographs taken at 40×). The bar graph indicates the result of DNA quantification (ng/mL). The vertical line depicts the standard deviation. **p < 0.01 compared to the control group.

Donor neutrophils exposed to PE sera showed a marked NET production compared to control sera exposure (**Figure 4**). This increased NET formation was evaluated by both fluorescence microscopy with SYTOX green staining (fold increase of 2.24 vs. control, *p* < 0.01) and DNA quantification in the supernatant (17.26 ± 0.52 ng/mL for PE-NETs vs. 13.61 ± 0.47 ng/mL for C-NETs, *p* < 0.01).

### PE-NETs induce a proinflammatory phenotype in endothelial cells in culture

**Figure 5:**
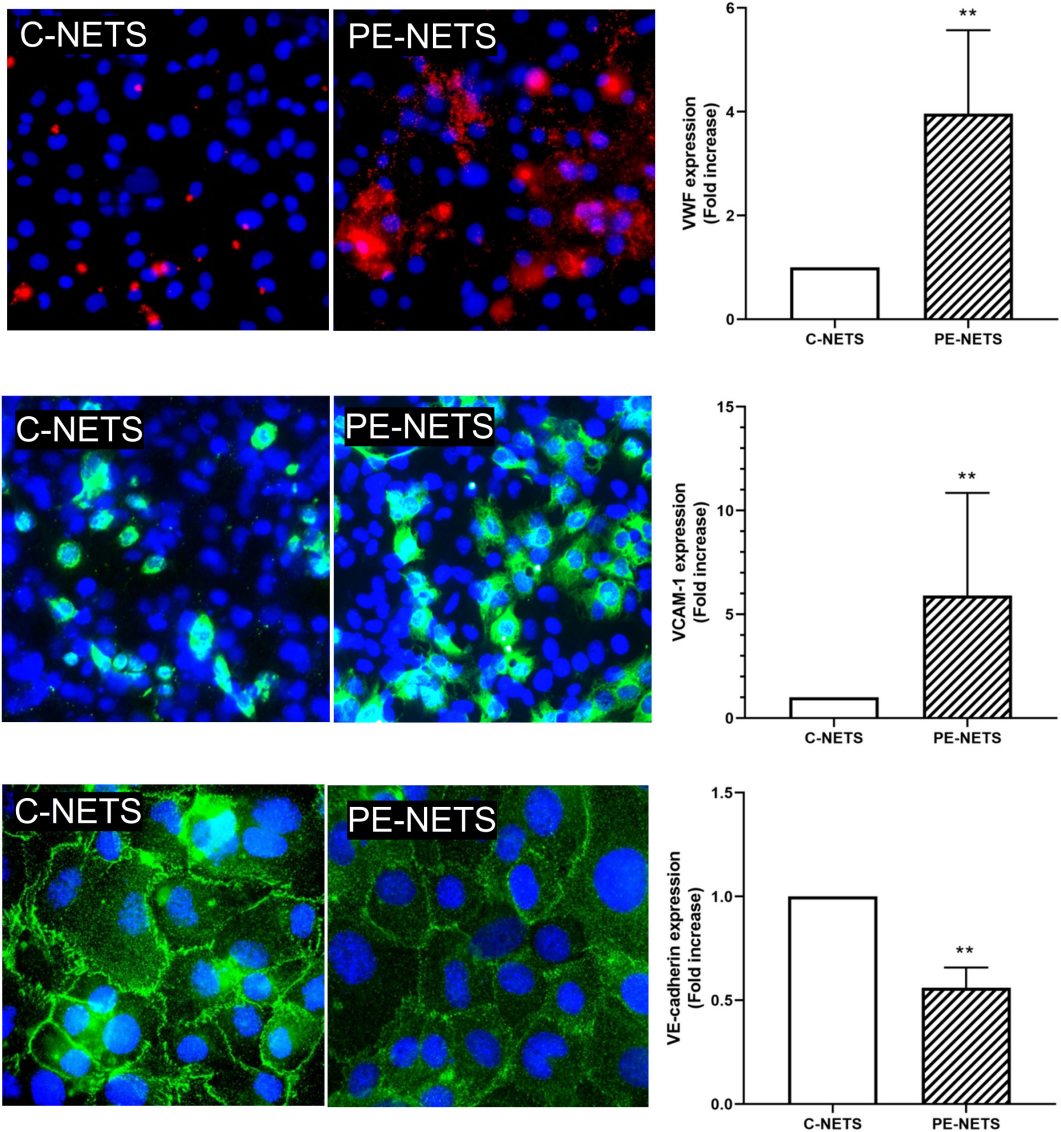
PE-NETs induce endothelial damage in the *in vitro* model compared to control NETs. Representative microscopy images of VWF (red, 40× micrographs), VCAM-1 (green, 40× micrographs), and VE-cadherin (100× micrographs) on endothelial cells (4′,6-diamidino-2-phenylindole-stained nuclei, blue) induced by exposure (48 h) to C-NETs and PE-NETS. The bar indicates the average fold increase compared to control. The vertical lines indicate the standard deviation. **p <  0.01 compared to the control group.

A prothrombotic state was observed in ECs incubated with PE-NETs compared to the exposure to C-NETs triggered by an increase of VWF release (fold increase of 4.0 ± 1.6 vs. C-NETs, *p* < 0.01). Moreover, ECs incubated with PE-NETs showed higher expression of VCAM-1 on cell surface compared to C-NETs (fold increase of 5.9 ± 1.2 vs. C-NETs, *p* < 0.01), while the expression of VE-cadherin was significantly lower in ECs incubated with PE-NETs compared to C-NET incubation (fold increase 0.5 ± 0.1 vs. C-NETs, *p* < 0.01) (**Figure 5**). No differences were observed between groups in ICAM-1, eNOS, and ROS biomarkers (*p* > 0.05) (**Supplementary Figure 2**).

### PE-NETs produce an increase in lytic complex C5b-9 deposition on ECs

**Figure 6:**
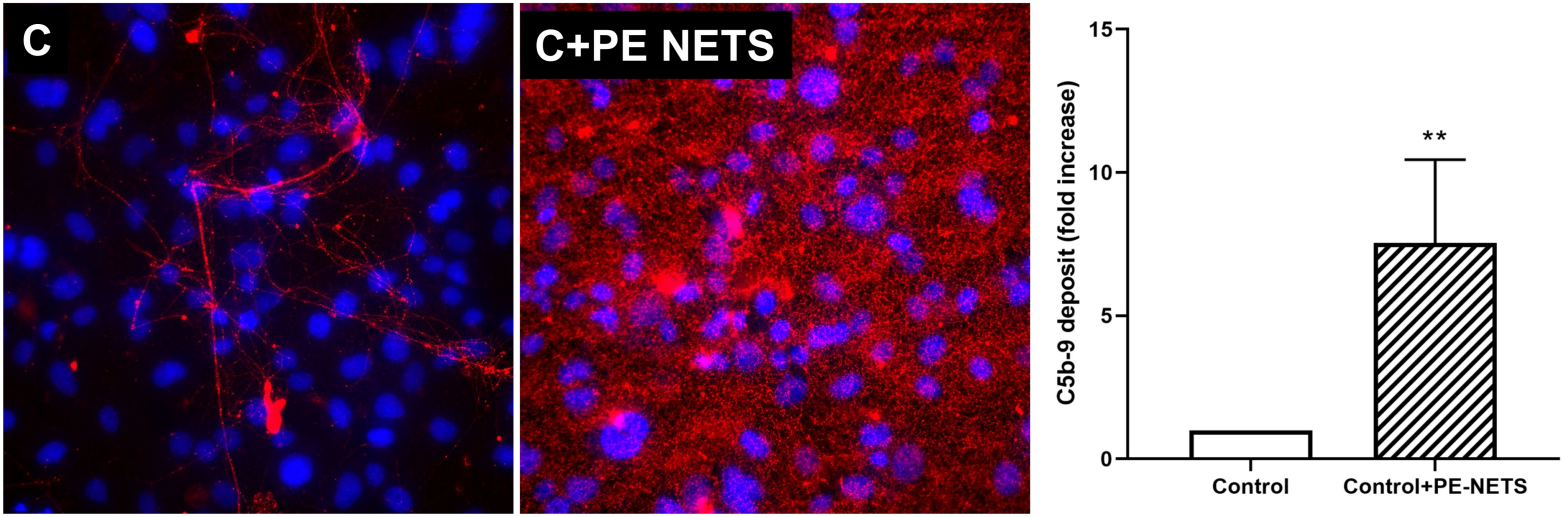
PE-NETs activate the complement system with an increase of the lytic complex C5b-9. Representative microscopy image (40×) of C5b-9 deposit stained with red fluorochrome on endothelial cells (4′,6-diamidino-2-phenylindole-stained nuclei, blue) induced by exposure (4 h) to control activated plasma **(C)** and this condition supplemented with PE-NETs (C+PE-NETS). The bar indicates the average fold increase compared to control. The vertical lines indicate the standard deviation. **p <  0.01 compared to the control group.

Complement function was evaluated through the quantification of the lytic complex C5b-9 deposition on ECs. These deposits were significantly triggered by PE-NETS. C5b-9 fold increase was 7.5 ± 2.9 in ECs incubated with PE-NETs compared to ECs incubated with control activated plasma (*p* < 0.01) (**Figure 6**).

## Discussion

The aim of the present study was to explore the contribution of EVs and NETs in the endothelial damage associated to PE using an *in vitro* model, and to explore their role as potential triggers of the complement system dysregulation described in these patients. Our results suggest that both elements play a pathogenic role in the endothelial phenotype described in PE, but activating different mechanisms. Moreover, NET overproduction in PE significantly contributes to complement system dysregulation, whereas no effect could be attributed to EVs.

Nowadays, no curative treatment has been established in PE, and the unique solution is pre-term labor to preserve mother and fetus health. Therefore, this disease is postulated as one of the most important complications during pregnancy. This pathology is intimately related to the endothelium. Although the PE patients of this study exhibited a slight overweight, its role in endothelial damage observed in this manuscript was discarded because none of these patients could be classified as obese. Our group has previously described high levels of soluble endothelial injury biomarkers such as VCAM-1 and VWF in these patients, and an increase of ICAM-1 and VWF expression in *in vitro* endothelial damage model in PE (30, 38). In addition, the overactivation of the complement system evaluated through an increase of C5b-9 deposition in ECs (37) has been proved in this pathology. Then, although endothelial damage and complement system dysregulation have been extensively demonstrated in this complication, the underlying mechanisms connecting these entities are unclear.

An abnormal placentation in the first trimester of pregnancy together with an imbalance of angiogenic factors (sFLT1 and PIGF), a pathophysiological immune activation (39), and the systemic activation of ECs of the maternal small arterioles in the late second or third trimester of pregnancy trigger a maternal endothelial crisis in PE. Moreover, there are some data relating this endothelial damage with placental-derived EVs (36); for instance, vasoconstriction and vascular endothelium damage observed in PE could be prevented and protected, respectively, by blocking the uptake of PE placenta-derived EVs (40). EVs released from the placenta to the blood exhibit a predominant role in paracrine and endocrine communication acting as homeostatic regulators in healthy pregnancies, but they also exhibit a potential implication in PE development (41–43). EVs contain proteins, mRNA, lipids, etc., surrounded by a lipid bilayer (44), and there are some studies suggesting differences between healthy pregnancy and PE-EVs (45). In our analysis, PE-EVs were not different from control EVs regarding size and concentration, but did present significant differences in their phenotype, especially in CD63, CD9, CD42a, and CD81 expression on their surface.

Both CD63 and CD9 are membrane markers expressed by platelets and immune cells that were found significantly lower in PE-EVs. CD63 has been proposed as a predictive PE biomarker (46) as it is increased in platelets from PE patients in the early stages of the complication but, to our knowledge, no evidence of CD63 and CD9 presence in PE-EVs has been described so far. Interestingly, we also found an increase of CD81 in PE-EVs compared to C-EVs, and this marker seems to be intimately related to PE pathogenesis (47). CD81, a member of the tetraspanin superfamily that plays significant roles in cell growth, adhesion, and motility, is significantly upregulated in sera from patients with early-onset severe PE. In addition, the exposure of ECs to a high dose of exogenous CD81 resulted in interrupted angiogenesis and EC activation (48). Regarding the detected lower expression of CD42a (or GPIX) in PE-EVs, another study observed a similar tendency but in platelets from PE pregnancies. This biomarker reflects severe PE progress and may be involved in its pathogenesis (49). Together with CD42a, CD29 and CD41b decrease was also observed in PE EVs. The EV composition described here could be responsible for their effect on the endothelial damage biomarkers evaluated in the present study.

In our *in vitro* model, the addition of PE-EVs to ECs in culture increased VWF and VCAM-1 expression and ROS production to similar levels to those observed when cells were exposed directly to PE sera, promoting the prothrombotic state and inflammatory phenotype previously described in these patients (50, 51). This increase in ROS production could activate NETosis as previous studies suggested (52) and be an additional mechanism to the presence of EVs and cytokines that activate neutrophils in a dose- and time-dependent manner (11). In contrast, PE-EVs alone did not induce a direct change in complement deposition. As expected, the exposure of cells to depleted-PE sera resulted in a milder effect on the endothelium, pointing to the deleterious effect of EVs. However, both VWF and ROS levels did not reach control levels in this condition, suggesting complementary mechanisms not related to EVs in the induction of the endothelial damage associated with PE.

PE sera dramatically increased NET production and release compared to control sera, in concordance with other studies (53). This phenomenon could be attributed to sera composition and maybe to the high interleukin levels, among other factors already reported in PE pregnancies. Like EV effects, PE-NETs also promote a prothrombotic and inflammatory phenotype state triggered by an increase of the VWF. NETs and VWF directly interact on the vascular wall and present a synergic effect promoting both the prothrombotic and proinflammatory phenotypes observed in PE. This proinflammatory phenotype was reflected in the detected overexpression of VCAM-1 in cells exposed *in vitro* to PE-NETs, in concordance with previous studies reporting that NETs increased VCAM-1 mRNA and protein expression in a time- and concentration-dependent manner (54). Although PE-NETs did not play a significant role in the induction of oxidative stress in our *in vitro* assays, ROS produced by EVs may be crucial for NET formation (55, 56). ROS overproduction found in PE, and triggered in our experiments by EVs, among other molecules, present the ability to activate transcription factors, such as NF-kB and AP-1 (57), promoting an overexpression of adhesion factors such as VCAM-1, and increasing IL-6 and IL-8 production (11, 48). This cytokine release is known to induce chemotaxis and neutrophil activation (58, 59), which, in turn, increases NET release (60).

Together with endothelial dysfunction and oxidative stress in PE, complement system dysregulation has been described as one of the most important pathophysiological mechanisms of this pregnancy complication (61, 62). Some evidence of the uncontrolled activation of the complement system in PE is the increment in C5b-9 membrane attack complex (MAC) deposition on ECs in *in vitro* assays (37), together with an increase of membrane attack complex at sites of villous injury in PE placental sections (63) and an increase in urinary excretion of C5b-9 (64) in these patients. The present study evidenced the direct effect of NETs in the overactivation of the complement system, causing a dramatic increase of C5b-9 deposition on ECs *in vitro* when added to the cell culture. Complement activation occurs not only on neutrophil membrane but also on released NETs (65), as NETs can directly activate an alternative complement pathway though properdin, factor B, and C3 (66, 67). Our results add new evidence to this intimate interaction between NETs and the complement system. Moreover, the potential linkage of severe PE to the most central complement gene, C3 (68, 69), makes this physical interaction between NETs and the complement system of interest for further investigations. In addition, it is known that the presence of complement split products, such as C3a and C5a (70), also contributes to hypertension and angiogenic imbalance in PE (71, 72). The terminal phase may be crucial in the management of PE, as complement inhibitors, such as anti-C5 (73) or antagonists of C5a receptor, reverted the angiogenic imbalance, prevented growth restriction and hypertension, and rescued pregnancies in an animal model (74). Moreover, the anti-C5 compound eculizumab has been used in pregnant women with satisfactory results (75–77). While the inhibition of the complement system may be a new treatment for PE, EVs and NETs may be a new target to improve the management of these patients.

## Conclusion

In conclusion, the present study demonstrates the role of both EVs and NETs as endothelial damage and complement dysregulation factors in PE. While EVs specifically activated oxidative stress, NETs could be the main factor responsible for the complement system overactivation. Our data suggest that EVs and NETs could be postulated as two critical elements participating in the pathophysiology of PE. Targeting the involved pathways by novel treatments that block the effect of EVs and NETs on ECs must be further explored in the management of PE. These treatments may potentially prolong the pregnancy and reduce maternal and perinatal complications.

## Data Availability

The original contributions presented in the study are included in the article/**Supplementary Material**. Further inquiries can be directed to the corresponding author.
