# Supplementary material for: Circulating extracellular vesicles and neutrophil extracellular traps contribute to endothelial dysfunction in preeclampsia
Source: Front Immunol. 2024 Dec 13;15:1488127. doi: 10.3389/fimmu.2024.1488127 (PMC11671372; doi:10.3389/fimmu.2024.1488127)

SUPPLEMENTAL DATA

Supplemental Figure 1. The exposure of endothelial cells to PE-EVs does not modify the eNOS, ICAM-1 and VE-Cadherin expression of dysfunction endothelial markers. Representative microscopy image (40X) of eNOS, ICAM-1 and VE-Cadherin (100X) (red: ICAM-1; green: eNOS and VE-Cadherin) on endothelial cells (4′,6-Diamidino-2-phenylindole–stained nuclei, blue) induced by exposure to control, preeclampsia, depleted preeclampsia (withput PE-EVs), and control sera pool supplemented with PE-EVs. The vertical bar indicates the average fold increase compared to control. The vertical line in bar indicates the standard deviation.

Supplemental Figure 2. PE-NETs do not contribute to oxidative stress in *in vitro* model with endothelial cells compared to control NETs. Representative microscopy images of eNOS, ICAM-1 and ROS (green: ROS (40X micrographs), VE-Cadherin (100X micrographs); ICAM-1 (40X micrographs) on endothelial cells (4′,6- Diamidino-2-phenylindole–stained nuclei, blue) induced by exposure to C-NETs and PE-NETS. The bar indicates the average fold increase compared to control. The vertical lines indicate the standard deviation.

Supplemental Figure 1

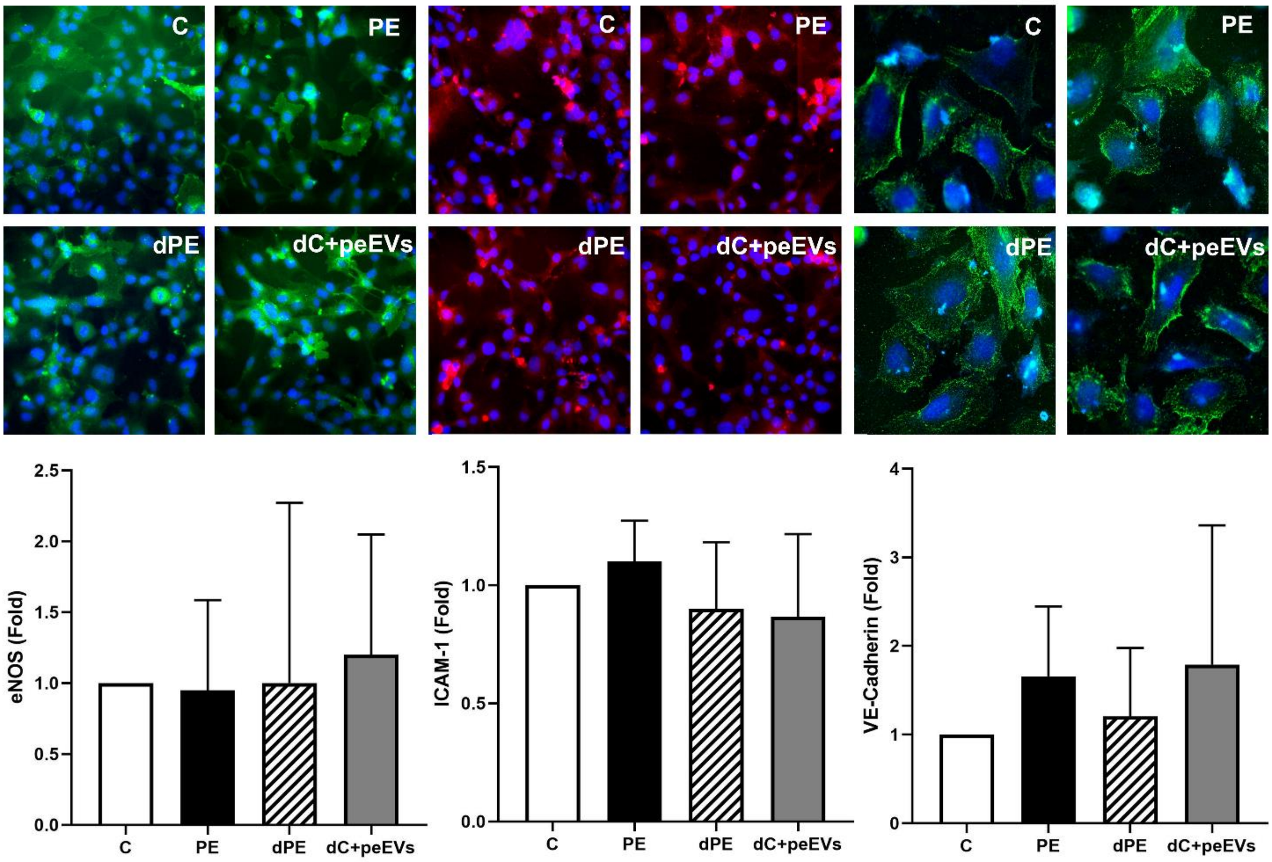

Supplemental Figure 2

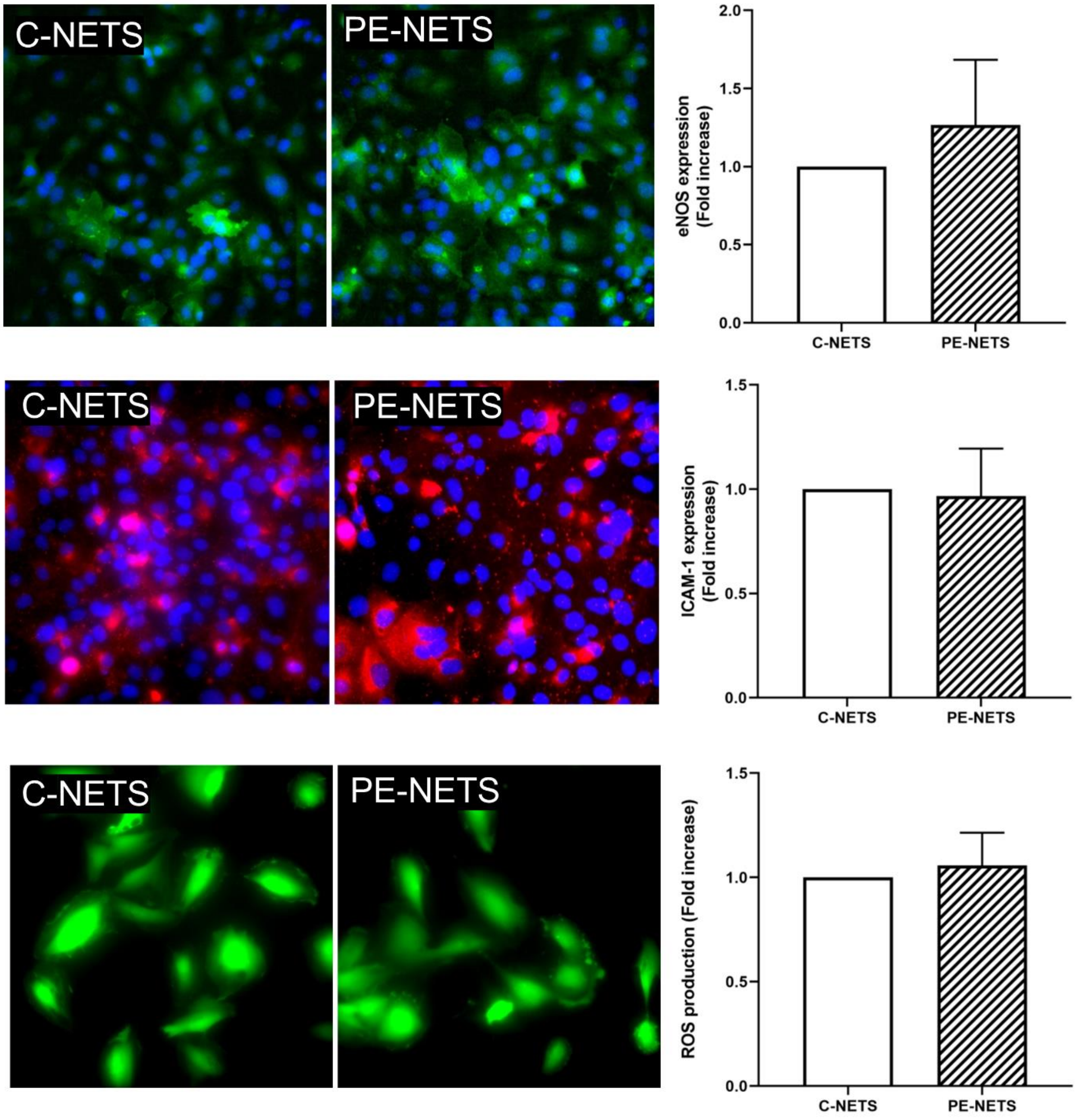

Supplement: Supplementary file 1 [file DataSheet1.pdf]
